# Pain Assessment in Neurodegenerative Diseases

**DOI:** 10.1155/2016/2949358

**Published:** 2016-06-28

**Authors:** Marina de Tommaso, Lars Arendt-Nielsen, Ruth Defrin, Miriam Kunz, Gisele Pickering, Massimiliano Valeriani

**Affiliations:** ^1^Neurophysiopathology of Pain Section, SMBNOS Department, Bari Aldo Moro University, Bari, Italy; ^2^Center for Sensory-Motor Interaction, School of Medicine, Aalborg University, Aalborg, Denmark; ^3^Department of Physical Therapy, Sackler Faculty of Medicine, Tel-Aviv University, Tel-Aviv, Israel; ^4^Section of Gerontology, Department of General Practice, University Medical Center Groningen, Groningen, Netherlands; ^5^CHU Clermont-Ferrand, Centre de Pharmacologie Clinique, 63003 Clermont-Ferrand, France; ^6^Inserm, CIC 1405, Neurodol 1107, 63003 Clermont-Ferrand, France; ^7^Division of Neurology, Ospedale Pediatrico Bambino Gesù, IRCCS, Rome, Italy

In recent years neurodegenerative diseases deserved growing attention for the progressive lengthening of human survival and the increasing prevalence of age-related brain disorders. Increasing competence is required by clinicians in regard to the different aspects of these chronic disabling disorders, as psychiatric and cognitive aspects, motor impairment, and pain. Diseases as dementia and motoneuronal and extrapyramidal disorders are opening a new scenario on pain syndromes, which represent an underestimated problem as the link between pathology and the pain experience is most often difficult to establish. This special issue was hosted by a panel of pain specialists around Europe and Israel, who shared common scientific interests on different aspects of pain, being particularly involved in researches on pain assessment and diagnosis in neurodegenerative diseases and especially dementia.

The issue contains a comprehensive review on pain aspects in common and rare neurodegenerative disorders where pain has recently emerged as a frequent symptom, although main treatments are exclusively focusing on cognitive or motor impairment and hence leave pain out of the guidelines. In Alzheimer disease, pain symptoms are not clearly expressed, so the few clinical scales actually used and in course of validation have the special aim to recognize features of sufferance. In Parkinson's disease and amyotrophic lateral sclerosis, pain has been described as a frequent associated condition, while few specific scales were employed and pain treatment is not guided by any quantitative assessments. In the review, the search for studies assessing the frequency and the clinical features of pain in these syndromes leads to the conclusion that it is a poorly studied symptom, despite its potential impact on the outcome of the diseases and main implication on the patients' quality of life. Up to now the available studies on the pathophysiological basis of pain in these disorders are inconclusive, and only few indications are present in regard to guiding the pharmacological approaches. The review outlined the need of a more focused attention by clinicians and researches on this emerging problem in the management of neurodegenerative diseases and the use of quantitative tools to evaluate the pain symptoms.

In addition, some original articles have also been included in this special issue to support this concept.

Since the first reports of altered pain responses in patients with cognitive impairment were published, the question has arisen, whether there might be certain cognitive domains (e.g., memory, attention, and executive functioning) that might best explain these alterations in pain responses. Kunz et al. [1] previously investigated this in a group of patients suffering from either mild cognitive impairment or dementia and found that “executive functioning” was the cognitive domain that best explained dementia-related alterations in pain responsiveness. In the present special issue, J. M. Oosterman et al. followed this approach and investigated which role executive functioning plays in explaining variations in self-report and facial expression to pain in a group of 52 older individuals. Facial expression and self-report were assessed in response to pressure pain of different intensities and these pain responses were then related to different domains of cognitive functioning. The authors could show that executive functioning (especially cognitive inhibition) indeed played an important role in explaining variations in pain responsiveness in older individuals. The poorer the ability for cognitive inhibition was, the stronger the facial expressions of pain across pain intensities were. Or in other words, impairment in executive functioning leads to a more pronounced communication of pain via the face. This study highlights the need to include tests of cognitive functions when assessing pain in older individuals.

T. F. Habiger et al. examined the complex relationship between pain, neuropsychiatric symptoms, and agitation in people with dementia. The authors performed a cluster randomized controlled trial and showed that in nursing homes patients with dementia exhibited reduced agitation, aberrant motor behaviour, and psychosis when they received pain treatment. This important trial suggests that pain is linked to psychosis and agitation among patients with dementia. In addition, the authors showed that opioid analgesics did not increase the prevalence of hallucination or delusion. These observations are particularly pivotal to provide adequate pain care in this vulnerable population.

M. W. de Vries et al. provided a good example of the difficulties of pain assessment in patients with cognitive impairment and their possible solution. They studied 153 patients with moderate to severe cognitive impairment by using the Orofacial Pain Scale for Non-Verbal Individuals (OPS-NVI). The “chewing” subscale was demonstrated to be reliable in assessing orofacial pain by means of the mere observation of video clips. Orofacial pain can be extremely frequent in elderly patients who have lost the capability to express it. Therefore, the development of behavioural scales able to reveal pain in these patients is mandatory to improve their quality of life. Of course, these tools need to prove their efficacy in large samples of patients, such as that investigated in this study.

Finally, one study by M. de Tommaso et al. reports original data on pain assessment in a rare disease such as Huntington's disease (HD). In HD motor symptoms as dystonia and hyperkinesis may be a potential cause of pain, but this has been only rarely reported in such disorder, suggesting a substantial dysfunction in pain expression. The present study reports the results of the application of a neurophysiological method as laser evoked potentials (LEPs) in early HD patients and genetically predisposed subjects in a presymptomatic phase. The LEPs showed clear abnormalities in both patients and those presymptomatic subjects who approached clinical onset, suggesting that the dysfunction of pain pathways is an early phenomenon which can influence sensory motor integration and the global outcome of the disease.

In conclusion, this special issue adds a small but potentially useful piece to the puzzle of pain expression and pain treatment in patients with cognitive and communication disorders, contributing to the understanding and management of the complex clinical aspects of pain in neurodegenerative diseases, a field which should receive more attention in the future.

*Marina de Tommaso* *Lars Arendt-Nielsen* *Ruth Defrin* *Miriam Kunz* *Gisele Pickering* *Massimiliano Valeriani*
